# Gut Microbiota-Bile Acid Crosstalk in Diarrhea-Irritable Bowel Syndrome

**DOI:** 10.1155/2020/3828249

**Published:** 2020-11-12

**Authors:** Kai Zhan, Huan Zheng, Jianqing Li, Haomeng Wu, Shumin Qin, Lei Luo, Shaogang Huang

**Affiliations:** ^1^The Second Clinical College of Guangzhou University of Chinese Medicine, Guangzhou 510000, China; ^2^Department of Gastroenterology, The Second People's Hospital of China Three Gorges University, Yichang 443000, China

## Abstract

The occurrence of diarrhea-predominant irritable bowel syndrome (IBS-D) is the result of multiple factors, and its pathogenesis has not yet been clarified. Emerging evidence indicates abnormal changes in gut microbiota and bile acid (BA) metabolism have a close relationship with IBS-D. Gut microbiota is involved in the secondary BA production via deconjugation, 7*α*-dehydroxylation, oxidation, epimerization, desulfation, and esterification reactions respectively. Changes in the composition and quantity of gut microbiota have an important impact on the metabolism of BAs, which can lead to the occurrence of gastrointestinal diseases. BAs, synthesized in the hepatocytes, play an important role in maintaining the homeostasis of gut microbiota and the balance of glucose and lipid metabolism. In consideration of the complex biological functional connections among gut microbiota, BAs, and IBS-D, it is urgent to review the latest research progress in this field. In this review, we summarized the alterations of gut microbiota in IBS-D and discussed the mechanistic connections between gut microbiota and BA metabolism in IBS-D, which may be involved in activating two important bile acid receptors, G-protein coupled bile acid receptor 1 (TGR5) and farnesoid X receptor (FXR). We also highlight the strategies of prevention and treatment of IBS-D via regulating gut microbiota-bile acid axis, including probiotics, fecal microbiota transplantation (FMT), cholestyramine, and the cutting-edge technology about bacteria genetic engineering.

## 1. Introduction

Irritable bowel syndrome (IBS) is the most common functional bowel disease, which can be categorized into diarrhea-predominant IBS (IBS-D), constipation-predominant IBS (IBS-C), mixed diarrhea and constipation IBS (IBS-M) and unspecified IBS (IBS-U), mainly symptoms including abdominal pain accompanied by increased defecation, loose stool, or mucus, without obvious organic abnormalities [1, 2]. The worldwide prevalence of IBS is 11.2%; in recent years, the number of patients is increasing, especially the number of young people, which makes the consumption of medical resources for diagnosis and treatment of IBS huge [3, 4]. The disease is mainly treated symptomatically, but it is easy to recur; therefore, patients are worried about suffering from malignant diseases, bearing a huge psychological burden, even causing mental and psychological disorders such as anxiety or depression, and the quality of life is greatly reduced [1].

The pathogenesis of IBS involves extensive and complex disturbances in the gut microbiota-bile acid metabolic axis [5]. Gut microbiota refers to various symbiotic bacteria and other microorganisms growing in the gastrointestinal tract [6, 7]. Under the interaction of host and microorganism, a lot of metabolic substances are produced, including BAs, choline, neurotransmitters, short-chain fatty acids (SCFAs), and other signaling factors and energy substrates, which are involved in gastrointestinal inflammation and carcinogenesis, liver disease, metabolic syndrome, IBS, and chronic diseases [8, 9]. BAs are synthesized in the liver, converted from the primary bile acids to secondary bile acids in the intestine where the microbiota make a significant impact on the process such as deconjugation and dihydroxylation. At the same time, the process will affect the size of the bile acid pool, resulting in the occurrence of various diseases [10]. In addition, BAs are not only for inhibiting the growth of gut microbiota and further destroying the stability of intestinal microecology in IBS patients due to excessive generation of cholic acid (CA) and deoxycholic acid (DCA) but also as ligands for the FXR and TGR5, and for inhibiting fibroblast growth factor (FGF) 19, which are related to the pathogenesis of IBS-D [11–14].

In the past few years, most previous studies have paid attention to the field of gut microbiota-bile acid axis in gastrointestinal carcinogenesis and inflammation; few have specifically focused on IBS-D. In this review, we discuss the effects of BAs, gut microbiota, and their interactions on IBS-D depending on current evidence from clinical and animal experiments, and present the potential future directions for the prevention and treatment of IBS-D by targeting the gut microbiota-bile acid axis.

## 2. Gut Microbiota Dysbiosis and IBS-D

It is the intestine that has the most number of species and densely populated habitat of microorganisms [15]. There are trillions of microorganisms colonized in the human body, which not only maintain our health but also bring us various diseases [8, 16]. In 2011, researchers found that people with IBS had altered gut microbiota via fecal analysis, which opened up possibilities for diagnostic tests and treatments [17]. Soon afterwards, a growing number of studies supported the view that the number and composition of the microbial community in feces and intestinal mucosa of IBS-D patients are different. For example, compared to healthy individuals (8.4 × 10^8^ colony-forming units [CFUs]/g feces), IBS-D patients have obviously lower concentrations of aerobic bacteria ((1.4 × 10^7^CFUs)/g; *P* = 0.002) [18]. Moreover, the recent analysis of fecal samples shows that the most main phyla of microbiota in IBS-D patients were Bacteroidetes (64.64%, vs. healthy controls (HCs) 56.43%), Firmicutes (26.14%, vs. HCs 35.97%), Fusobacteria (5.18%, vs. HCs 1.39%), and Proteobacteria(3.73%, vs. HCs 5.66%), which indicated the percentage of rich phyla Firmicutes was obviously reduced and Bacteroidetes was raised in IBS-D patients [19, 20]. In addition, some bacteria, including Escherichia coli, Pseudomonas aeruginosa, Staphylococcus aureus, and Enterococcus faecalis, could inhibit the growth of intestinal probiotics, such as Bifidobacterium and Lactobacillus [21], which could accelerate the vicious cycle of intestinal flora imbalance that leads to IBS-D. At the genus level, Lachnospira, Ruminococcaceae_UCG003, Lactobacillus, Enterococcus, Weissella, Lachnospiraceae_UCG-010, Oxalobacter, Parasutterella, Turicibacter, and Oceanobacillus were significantly decreased, while Faecalitalea was increased in IBS-D patients when compared with HCs (*P* < 0.05) [19]. But Carroll et al. [22] found an obvious reduction in the concentrations of the Fecalibacterium genus in IBS-D patients when compared to HCs. The difference between them may be caused by different original regions of patients, one is from China, the other is from the USA, which tells us the changes in composition and diversity of the gut microbiota in IBS-D patients may be different, even to be the opposite outcome. The possible reason lies in the small sample size and detection error. Of course, it also reminds us that the response of IBS-D patients in different areas to the same probiotic preparation may be completely different, which depends on the changes of gut microbiota of local patients.

In addition, probiotics play a vital role in the treatment of IBS. In 2018, an open-label, prospective study has shown, compared with patients with non-IBS-D (*n* = 15), treatment with commercial probiotics for 30 days could obviously improve bowel function satisfaction in patients with IBS-D (*n* = 11) (*P* = 0.05) [23]. A double-blind randomized placebo-controlled pilot clinical study indicated that IBS patients' clinical symptoms, including diarrhea, abdominal pain, bloating, stool frequency, and vomiting, could be obviously relieved after treating with B. coagulans MTCC 5856 at a dose of 2 × 10^9^cfu/day [24]; the effect of it could be involved in producing SCFAs, acetate, butyrate, and propionate [25]. Sjögren et al. [26] observed fecal microbiota transplantation increased gut microbiota diversity (Verrucomincrobia and Euryarchaeota) in patients with IBS-D and significantly improved symptom and quality of life. It is also explained from the side that gut microbiota has a vital relation with IBS-D according to the treatment of probiotics, fecal microbiota transplantation, and rifaximin on IBS-D.

So far, the pathogenesis of IBS is not completely clear, but recent researches have shown that gut microbiota could affect enterochromaffin (EC) cells and thus change the expression levels of 5-hydroxytryptamine (5-HT) [27–29] which can increase visceral sensitivity and trigger IBS-D [30]. At the same time, the metabolites of Clostridium could upregulate the tryptophan hydroxylase (Tph) gene expression in EC cells to promote 5-HT production [31]. A study suggested that Clostridia-rich microbiota of IBS-D could suppress the expression levels of FGF19 in the intestine to promote liver synthesizing and secreting more bile acids [31], which is a process of the inhibition of BA formation in the negative feedback mechanism [32]. Moreover, SCFA, the main metabolites of intestinal flora, can reduce the amount of E. coli, increase the production of lactobacilli, and inhibit the permeability of the intestinal mucosa to protect the function of the intestinal mucosal barrier [33]. According to the current literature, most of the studies focus on the relationship between IBS-D and the number and structure of intestinal flora, while the mechanism of a specific bacteria on IBS-D is less.

## 3. Bile Acid-Gut Microbiota Axis in IBS-D

### 3.1. Bile Acid Synthesis, Transport, and Metabolism

**Figure 1:**
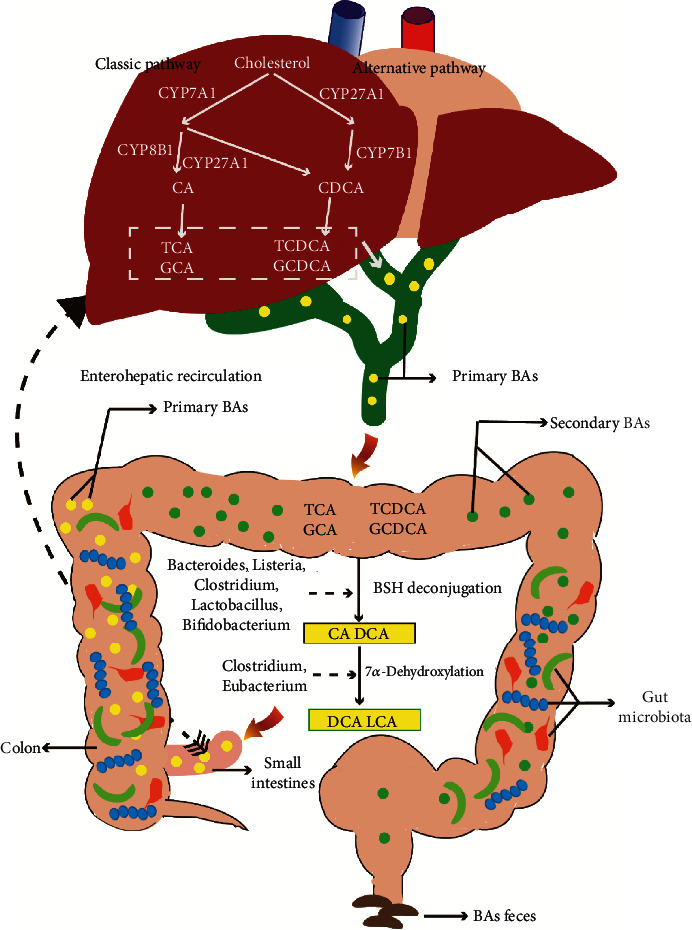
Bile acids biosynthesis, metabolism, and its relationship with gut microbiota. In humans, bile acids (BAs) mainly consist of primary BAs and secondary BAs. The primary BAs include CA and CDCA, which are synthesized by cholesterol in hepatocytes through the classical pathway and the alternative pathway. The secondary BAs mainly include DCA and LCA, which are converted from primary BAs by gut microbiota. The major genera of gut microbiota take part in secondary BA production which includes Lactobacillus, Bifidobacterium, Clostridium, Listeria, Bacteroides, and enterococcus in deconjugation and Eubacterium and Clostridium in 7*α*-dehydroxylation. Abbreviations: CA: cholic acid; CDCA: chenodeoxycholic acid; DCA: deoxycholic acid; LCA: lithocholic acid; TCA: taurocholic acid; TCDCA: taurochenodeoxycholic acid; GCA: glycocholic acid; GCDCA: glycochenodeoxycholic acid; CYP7A1: cholesterol 7*α*-hydroxylase; CYP8B1: sterol 12*α*-hydroxylase; CYP27A1: sterol 27-hydroxylase; CYP7B1: oxysterol 7a-hydroxylase.

BA synthesis from cholesterol takes place in the hepatocytes and occurs through two different pathways. One is the classical pathway, which is regulated by 3 cholesterol hydroxylase enzymes: mitochondrial sterol 27-hydroxylase (CYP27A1), cholesterol 7*α*-hydroxylase (CYP7A1), and sterol 12*α*-hydroxylase (CYP8B1) [16, 34]. More than 90% of the total BAs are synthesized by the classical pathway. The other is the alternative pathway, which produces chenodeoxycholic acid (CDCA). The process starts with the hydroxylation of the cholesterol by CYP27A1, and then, it is transformed by oxysterol 7*α*-hydroxylase (CYP7B1) [9, 16]. In the liver, the two most primary BAs (CDCA and CA) are converted to conjugated primary BAs (TCDCA/GCDCA and TCA/GCA) and secreted into the bile after they are conjugated to glycine or taurine [9, 35]. After the bile containing GCA/TCA and GCDA/TCDCA enters the intestine, they will be converted into secondary bile acids including DCA and lithocholic acid (LCA) in two major biotransformations: 7*α*-dehydroxylation and deconjugation (biochemical reaction process of bile salt hydrolase (BSH) hydrolyzes the conjugated BAs), which are the important processes for gut microbiota to affect the BA metabolism [36, 37]. The detailed process between them is shown in Figure 1.

### 3.2. Influence of Gut Microbiota on Bile Acids

Gut microbiota is a complicated ecosystem, which consists of over 1000 microbial species and 10^14^ cells, containing genes that are 150 times more than the human genome [34, 35]. According to the latest research, the ratio between microbial cells and human cells in the body is 1.3-2.3 : 1, not the previous ratio of 10 : 1 [38, 39]. In fact, over 90–99% of the microbial community in healthy humans and animals are mainly two phyla which are represented by Bacteroidetes and Firmicutes; the others are fewer members in Actinobacteria, Fusobacteria, Proteobacteria, and Verrucomicrobia [39–41]. In physiological condition, gut microbiota contributes to maintaining host health through the production of essential vitamins, food digestion, fighting pathogens, and molecular interaction with the host and plays a vital role in the maturation of the host digestive system. Of course, there are many kinds of microbiota among them which play a vital influence on the process of producing secondary BAs in the intestine, such as Listeria monocytogenes, B. vulgatus, Lactobacillus, Clostridium perfringens, Bifidobacterium, Bacteroides fragilis, and the genus Clostridium. The detailed relationship between these bacteria and IBS is shown in Table 1.

In humans, gut microbiota is involved in the secondary BA production via deconjugation, 7*α*-dehydroxylation, oxidation, epimerization, desulfation, and esterification reactions, respectively; two important reactions of them are deconjugation and 7*α*-dehydroxylation [36, 37]. The enzymatic hydrolysis of the C-24 N-acyl amide carried out by BSH in the small intestine is called as deconjugation [42, 43]. BSH activity can be highly expressed by commensal bacteria inhabiting in the small and large intestine, including Bacteroides, Clostridium, Lactobacillus, Bifidobacterium, Enterococcus, and Listeria [9, 16, 44–47]. Apart from deconjugation, BAs undergo additional biotransformation; it is the bacterial 7*α*-dehydroxylation that converts nearly all CA (with hydroxy groups at C-3, C-7, C-12) and CDCA into DCA and LCA in the colon, respectively [47, 48]. It has been confirmed that Clostridium and Eubacterium are involved in 7*α*­dehydroxylation. The 16S rRNA sequence analyses in recent years have shown the genus Clostridium is involved in the reaction, including C. hiranonis, C. scindens, C. hylemonae (Clostridium cluster XIVa), and C. sordellii (Clostridium cluster XI) [26, 42, 48–50]. But some research indicated that C. hylemonae (Clostridium cluster XIVa) was the only distinct members of Clostridium to undergo this reaction [42, 47, 51]. Therefore, further research is needed in the field of intestinal flora. Moreover, Eggerthella, Clostridium, Bacteroides, Peptostreptococcus, Ruminococcus, Eubacterium, and Escherichia participate in catalyzing epimerization and oxidation of the hydroxyl (OH) groups at C3, C7, and C12 [9, 52, 53]. At the same time, a research indicated, compared to the conventional mice (88 ± 1.7% relative to the total BA), germ-free mice hardly detected secondary BAs (1.8 ± 0.2%) in fecal samples, but the latter (86.8 ± 0.8%) had a higher proportion of conjugated BAs [54]. This is one of the strong evidence for the involvement of gut microbiota in deconjugation and dehydroxylation of bile acids.

### 3.3. Influence of Bile Acids on Gut Microbiota

BAs serve as environmental cues and nutrients to microbes, but they also have a direct antimicrobial on gut microbiota and can cause disease by regulating gut microbiota. A number of studies have suggested higher BA concentration exert cytotoxicity, causing apoptosis, inducing proinflammatory actions and DNA damage, producing necrosis, and involving functional gastrointestinal disorders (FGID) [55–58]. Some researches have revealed that DCA is one of the most effective antimicrobial bile, its bactericidal activity is 10 times than that of CA, and it can seriously inhibit the growth of gut microbiota such as lactobacilli, Clostridium perfringens, bifidobacteria, and Bacteroides fragilis [59, 60]. CA can decrease beneficial bacteria Roseburia, Lactobacillus, and Ruminococcus [61]. Feeding CA to rats raised the proportion of Firmicutes/Bacteroidetes, simplified the diversity of gut microbiota, and increased the growth of some microorganisms in the classes Erysipelotrichi and Clostridia which mainly includes the genus Allobaculum and the genus Blautia, respectively [12, 62].

The evidence indicated that nearly 68% of patients with IBS-D had increased total fecal BAs or bile acid malabsorption [55, 63]. In addition, the genes klotho B (KLB) and fibroblast growth factor receptor 4 (FGFR4) related to BAs make a significant influence on accelerating small intestinal or colonic transit in IBS-D [64, 65]. Dior et al. [66] found that primary BAs were obviously increased and secondary BAs were obviously decreased in IBS-D patients' serum and stool compared to healthy subjects. At the same time, the BA receptors TGR5 and FXR can make a vital influence on metabolic disorders and promote the production of secondary BAs in the gut [10]. In animal experiments, people found that DCA could induce colon net water secretion and have an excitatory effect on motility in the rat proximal colon [67, 68]; that is one of the reasons of IBS-D [69, 70]. Oral administration of CDCA could increase defecation frequency and accelerate colonic transit in a dose-dependent manner [69, 71, 72].

## 4. Bile Acid-Activated Receptors and Signals

BAs, acting as signaling molecules, also play a significant role in the human body.

**Figure 2:**
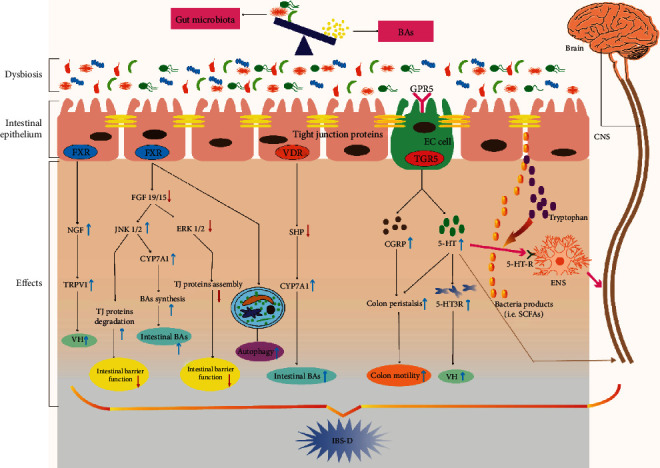
Summary of the possible signaling pathway between gut microbiota-bile acids axis and IBS-D. The metabolic disorder of the gut microbiota-bile acid axis could lead to the higher secretion levels of secondary BAs, which activate a series of signaling pathways in the intestinal epithelium, resulting in VH, damage of intestinal mucosal barrier function, increased intestinal motility, and increased intestinal bile acid excretion, thus promoting the occurrence of IBS-D. On the one hand, the reduced expression of FXR can not only upregulate the NGF/TRPV1 signaling pathway to cause VH but also downregulate FGF19/15 that can regulate the JNK1/2 and ERK1/2 signaling pathway to make an inhibition on intestinal barrier function and promote autophagy. On the other hand, the increased secondary BAs can activate TGR5 on EC cells to up-regulate the expression of 5–HT and CGRP, causing increased colonic motility. At the same time, 5-HT could upregulate the release of 5-HT3R to cause VH and transmit stimulus to the spinal cord, the process of which may be involved in the brain-gut interaction. Abbreviations: BAs: bile acids; FXR: farnesoid X receptor; NGF: nerve growth factor; TRPV1: transient receptor potential vanilloid 1; VH: visceral hypersensitivity; FGF19/15: fibroblast growth factor (FGF) 19/15; JNK: c-Jun N-terminal kinase; ERK: extracellular signal-regulated kinase; TJ: tight junction; CYP7A1: cholesterol 7*α*-hydroxylase; SHP: small heterodimer partner; TGR5: G-protein-coupled bile acid receptor 1; 5-HT: 5-hydroxytryptamine; 5-HT3R: 5-hydroxytryptamine 3 receptor; CGRP: calcitonin gene-related peptide; EC: enterochromaffin; SCFA: short-chain fatty acids; ENS: enteric nervous system; CNS: central nervous system; VDR: vitamin D receptor.

They could regulate various physiological functions via interaction with FXR, vitamin D receptor (VDR, NR1H1), TGR5, pregnane X receptor (PXR, NR1I2) and constitutive androstane receptor (CAR, NR1I3) [11–13, 73, 74], and cell signaling pathways such as extracellular signal-regulated kinase (ERK) and c-Jun N-terminal kinase (JNK) [75]. BAs can bind to different nuclear receptors and activate them in a rank order. For example, in most human liver and colon cell lines, FXR can be activated in a rank order of CDCA>DCA>LCA>CA. While the rank order of TGR5 is different, one is LCA>DCA>CDCA>CA for unconjugated BAs [76], the other is TLCA>LCA>GLCA>TDCA>DCA>GDCA>TCDCA>CDCA>GCDCA>TCA>CA>GCA for both conjugated and unconjugated BAs [77]. PXR and VDR are activated only by LCA. The following is a review of TGR5, FXR, and their related pathways (Figure 2).

### 4.1. Gut Microbiota-FXR Axis

The gut microbiota regulates FXR signaling via intervening BA metabolism and the production of secondary BAs. FXP is the main BA senor and highly expressed in the ileum and liver [78]. It takes part in many biochemical reaction processes, including regulating the bile acid homeostasis, lipid, and glucose metabolism [36]. In terms of bile acid synthesis in humans, it is tightly regulated by the negative feedback inhibition through FXR primarily by two downstream target genes, FGF19 (FGF15 in mice) in the ileum and small heterodimer partner (SHP) in the liver [79]. Studies have indicated that FXR and its two downstream target genes (SHP and FGF15) are highly expressed in the gut of the conventionally raised (CONV-R) mice when compared to germ-free (GF) mice [80, 81]. In turn, the circulating FGF19 decreases the expression of liver cholesterol 7*α*-hydroxylase and BA synthesis [82]. Moreover, the fecal BA excretion and hepatic BA synthesis in mice have been increased by changing the gut microbiota profiles using probiotics, the mechanism of which has a close connection with the inhibition on the enterohepatic FXR–FGF15 axis [83]. Sayin et al. [80] found that the expression levels of SHP and FGF15 induced by microbiota were completely inhibited in FXR^−/−^ mouse ileum, which directly evaluates the influence of gut microbiota on the target downstream of FXR. The new research shows that Clostridia-rich microbiota can result in an excessive BA production by suppressing the intestinal FGF19 expression in IBS-D patients [14]. On the other hand, BAs make an indirect influence on gut microbiota via regulating the expression levels of FXR. It has been pointed out recently that FXR activated by BAs could upregulate some gene expression (such as Ang1, iNos, and Il18) to inhibit the bacterial overgrowth and mucosal injury in the gut [84].

With the deepening of research, more and more people also have realized the relationship between FXR and IBS-D. Recent researches have shown that FXR mRNA had higher expression levels in the rectosigmoid mucosa of IBS-D patients [85, 86], while Horikawa et al. [87] found it was also obviously increased in IBS patient's ileum, but not in the rectum, duodenum, and cecum. Given the difference of the study, we considered that the sample of Horikawa et al. was small (15 IBS patients, including 8 IBS-D patients, 7 IBS-M patients). So, further research needs to be done. In animal research, compared to wild-type mice (FXR^+/+^), Vavassori et al. [88] observed a mild to moderate cellular infiltration of the colonic mucosa lamina propria and increased mRNA expression of IL-1*β*, TGF*β*1, and TNF-*α* in FXR^−/−^ mouse colons. We can infer that the FXR gene ablation leads to the dysregulation of intestinal immunity and proinflammatory, which make a significant influence on the mechanism of IBS. In addition, visceral hypersensitivity is involved in IBS-D. Li et al. [89] demonstrated that visceral hypersensitivity induced by prolonged colonic BA stimulation was involved in the FXR/Nerve growth factor (NGF)/transient receptor potential vanilloid (TRPV) 1 axis. On the other hand, FXR also has a connection with the gut microbiota and autophagy. For example, FXR-deficient mice could increase the expression levels of Firmicutes and decrease the expression levels of Bacteroidetes [81], which is consistent with the changes of gut microbiota in IBS-D according to the recent study. Moreover, Lee et al. [90] found FXR could bind to shared sites in autophagic gene promoters to suppress autophagy in mice. In the gut-specific autophagy-related 5 knockout (Atg5^−/−^) mouse model, researchers found that the composition and richness of the gut microbiota were changed, with increasing Candidatus Athromitus and the Pasteurellaceae family and decreasing the Lachnospiraceae and Akkermansia muciniphila family [91]. Therefore, we could infer that the potential connections between autophagy and gut microbiota regulated by BA-activated receptors could exist, which may contribute to a profound influence on the pathological mechanism research of IBS-D. Of course, some researches pay attention to the FXR downstream target genes FGF19/15, which are involved in abnormal BA metabolism so as to IBS-D. FGF19/15 induced by FXR in the ileum binds to the FGF receptor 4 (FGFR 4)/*β*-klotho heterodimer complex in the liver, which actives the JNK1/2 and ERK1/2 pathways to inhibit the expression of the CYP7A1 gene [92, 93] to regulate BA metabolism. In IBS-D patients with excess total BA excretion in feces (≥10.61 *μ*mol/g), the concentration of serum FGF19 was decreased when compared with the healthy controls [14]. Moreover, Vijayvargiya et al. [94] found fasting serum FGF19 levels had good specificity and negative predictive value by testing 101 patents with IBS-D. Further researches indicated the activation of the JNK pathway could lead to the degradation of the tight junction protein, while the activation of the ERK1/2 pathway could promote the assembly of tight junction protein and repair the solidified intestinal mucosal barrier [95–100]. In addition, Dai et al. [101] confirmed that VSL#3 probiotics could activate the ERK signaling pathway to increase the expression levels of tight junction protein in vivo and in vitro so as to protect the epithelial barrier. We have previously discussed the important role of the intestinal mucosal barrier in IBS-D; according to these results, we consider that the gut microbiota-BAs-FXR-FGF19/15-JNK/ERK pathway may be an attractive potential mechanism for IBS-D.

### 4.2. TGR5

TGR5 is another BA-responsive receptor with high expression in the intestine L tissues, gallbladder epithelial cells, gallbladder smooth muscle cells, hepatic sinusoidal endothelial cells, Kupffer cells, and immune cells [76, 77, 102, 103]. It can be activated by the primary and secondary BAs, and it mainly sends its signal by increasing the intracellular concentrations of cyclic AMP (cAMP), resulting in stimulating the expression of downstream cAMP-dependent protein kinase A (PKA) [104]. Though the exact relationship between TGR5 and IBS-D is still unknown, we will review the most relevant pathological mechanism of IBS-D at present.

In the gastrointestinal tract, TGR5 induced by BAs protects the intestinal barrier function and reduces inflammation [105]. When compared to WT mice, TGR5^−/−^ mice had an abnormal morphology of the colonic mucous and an increased intestinal permeability [106] which might cause their raising susceptibility to IBS-D. Intestinal epithelium mainly consists of adherens junction, desmosome, and tight junction; among them, tight junctions are multiprotein complexes including claudins and occluding proteins. Moreover, zonula occludens 1 (Zo1) and Zo2 are significant to tight junction assembly and maintenance [107]. Fewer researches focus on the tight junction protein and TGR5 in IBS-D, while we can find a little literature about other diseases. For example, Abu-Farsak et al. [108] found there was a positive correlation between the expression of Claudin-2 and TGR5 in the esophageal tissue, and the expression levels of claudin-2 were significantly increased from normal squamous mucosa to columnar cell metaplasia, Barrett's esophagus, and low- and high-grade dysplasia to esophageal adenocarcinoma. On the other hand, Yang et al. [109] demonstrated that TGR5 activated by BAs could activate the JNK pathway. It was discussed above that the JNK pathway could lead to the degradation of tight junction protein [95–100]. Therefore, we could infer from these results that excessive BAs in the gut may induce the expression of TGR5, activate the downstream JNK signal pathway, increase the permeability of mucosa, and eventually damage the mucosal barrier. If so, the result is contradictory like the studies of claudin-2; it needs much more experiments to confirm. At the same time, the gut microbiota and their metabolic products could directly regulate the expression of tight junction protein to change the enteric mucosal permeability and, eventually, achieve the ability to maintain the integrity of the intestinal epithelial barrier [110, 111]. In turn, alternations in the composition and richness of the gut microbiota can also cause an increase in mucosal permeability and a damage to the intestinal epithelial barrier function [112].

Some researches indicated that TGR5 involved BA-induced gastrointestinal motility [113, 114]. Up to now, the action is stimulatory or inhibitory and still exists controversy. In vitro, Alemi et al. [115] reported that various TGR5 agonists could regulate peristalsis of colon full-thickness segments in wild-type (WT) mice, but there was no influence in TGR5^−/−^ mice. And in vivo, TGR5^−/−^ mice had slowed colonic transit, had reduced frequency of defecation, and had lower fecal water content compared with WT mice and TGR5-transgenic mice, even constipation. Moreover, TGR5-transgenic mice had accelerated colonic transit and increased 1.4-fold pellet excretion. In the colon, BAs activate TGR5 on enterochromaffin (EC) cells to upregulate the expression of 5–HT and on enteric neurons to upregulate the expression of calcitonin gene-related peptide (CGRP). TGR5 and CGRP could promote colon peristalsis [116]. But some studies have shown TGR5 could slow small intestinal motility and may serve in the “ileal brake,” a mechanism that slows the intestinal transit during digestion so as to improve the absorption of nutrients [117, 118]. Further evidence has shown that BA-induced TGR5 in colon epithelial may contribute to limiting the fluid secretion into the lumen in order to prevent its excessive loss from feces [117]. Consistent with their researches, Poole et al. [118] found the expression level of TGR5 mRNA was widely increased around the GI tract, particularly in the enteric ganglia, and prominently expressed by inhibitory motor neurons; its activation inhibited intestinal contractility and slowed gastric emptying and small intestinal transit. The effect may involve in the nitrergic mechanism, and/or G*α*_s_/cAMP pathway regulating muscle relaxation [118, 119]. Moreover, direct evidence suggests there is a connection between the TGR5 genotype variations (rs11554825) and small intestinal transit in IBS-D patients [65]. Therefore, from one aspect of these results, we may consider BAs-TGR5-(5-HT)/CGRF- accelerates the colon peristalsis axis and plays a vital role in IBS-D.

TGR5 mRNA is expressed on astrocytes and neurons in the mouse and human brains [120]. It is discussed above that TGR5 could upregulate the expression of 5–HT on EC cells in the GI tract [116], and there are 95% of the body's 5-HT stored in EC cells and enteric neurons, and only 5% in the central nervous system (CNS) [121]. The evidence has shown 5-HT is involved in the neuroendocrine pathway within the brain-gut-microbiome axis via the essential amino acid tryptophan (Trp) [122, 123]. Further research indicated the expression of plasma 5-HT and Trp levels in germ-free mice were prominently decreased compared to conventionally colonized mice [25, 124]. Moreover, current evidence has shown that microbiome regulated the CNS which occurs mainly via the neuroimmune and neuroendocrine mechanisms [125–127]. This process is involved in some microbially derived molecules, such as tryptophan metabolites, SCFAs, and secondary bile acids [25, 29, 125]. Therefore, we can see the complex relationship among intestinal microbiota, TGR5, and 5-HT in the brain-gut-microbiome axis from these results. In addition, Lacy et al. [3] show the disturbances in the brain-gut function were one of the most important mechanisms in IBS. A retrospective analysis has shown 5-HT and 5-hydroxytryptamine receptor 3 (5-HT3R) were both prominently expressed in the intestinal mucosa tissue of IBS-D patients when compared with healthy subjects [128]. At the same time, 5-HT3R antagonists contributed to suppress urgency, alleviate symptoms, and prolong the intestine transit in IBS-D, while 5-HT3R agonists could promote intestinal motility, and accelerated transit in IBS-C patients [129]. The possible mechanisms are the activation of 5-HT which promotes visceral hypersensitivity causing irritable bowel syndrome [130, 131]. So, with the emerging evidences that occurred, the brain-gut-microbiome axis may become a new hot topic in IBS.

## 5. Targeting Microbiota-Bile Acid Axis for the Treatment of IBS-D

### 5.1. Probiotics

Due to their widespread influence on intestinal cells and tissue and their vital role in many physiological processes, the microbiota-bile acid axis may contribute to providing a potential therapeutic direction in the treatment of IBS-D. A number of recent systematic reviews of the literature and meta-analyses have concluded that probiotics have a limited but significant therapeutic effect over placebo on IBS symptoms [132–134]. Probiotic VSL#3 consisted of 8 Gram-positive bacteria strains (1 species of Streptococcus thermophilus, 3 species of Bifidobacterium, and 4 species of Lactobacillus). Degirolamo et al. [83] show the evidence that VSL#3 probiotics could promote ileal BA deconjugation and fecal BA excretion and increase hepatic BA neosynthesis in vivo by downregulation of the gut-liver FXR-FGF15 axis. Moreover, probiotic VSL#3 makes a vital influence on decreasing visceral sensitivity in patients with IBS [135, 136], resetting colonic expression level of subsets of genes regulating inflammation and pain and reducing visceral pain perception in the murine model of IBS [137]. Some other studies indicated the L. acidophilus NCFM and L. johnsonii strain 100–100 could be involved in the BA metabolism owing to the ability of hydrolyzing bile salts [138, 139]. A randomized, double-blind, placebo-controlled trial in Vietnamese patients with unconstipated IBS indicated the new combination of Lactobacilli including L. salivarius, L. plantarum, and L. paracasei could relieve the abdominal symptoms [140]. The mechanisms of the effect of probiotics in IBS are only partially known. Researches show that the different effects of different strains of probiotics on IBS patients, such as Bifidobacterium longum subsp. longum NCC3001 (BL) could change brain activity and decrease depression scores [141]. Bifidobacterium lactis DN-173-010 could accelerate colonic transit in IBS-C patients [142], and Escherichia coli strain Nissle 1917 (EcN) could decrease the visceral pain caused by IBS [143]. According to the new systematic review, in which the data was conducted in Medline (PubMed) from 2014 to March 2019, it has more significant beneficial effects on improving IBS symptoms than using multistrain probiotics supplements for 8 weeks or more when compared with a monostrain probiotics supplement [144], but patients treated with probiotics had a higher incidence of any adverse event (relative ratios (RR) 1.21; 95% confidence interval (CI) 1.02-1.44) [145]. Therefore, the dose and duration of treatment of multistrain probiotic supplementation on IBS patients should be established via further long-duration randomized controlled trials (RCTs).

### 5.2. Fecal Microbiota Transplantation (FMT)

Fecal microbiota transplantation (FMT) may act as a potential therapy to treat IBS. Since the first RCT on FMT treatment for IBS has been started in Norway in 2017 [146], the exploration of FMT for IBS has never been stopped. In their study, 65% of the participants who received FMT treatment and 43% of the participants who received placebo for 3 months reduced more than 75 points of the IBS-severity scoring system (*P* = 0.049), which show that the treatment group had a better effect than the placebo group. Moreover, in 2019, Johnsen et al. also confirmed that IBS-related quality of life and fatigue in patients with nonconstipated IBS were significantly relieved after treating by FMT [147]. In 2020, a clinical study has shown the symptoms (diarrhea, bloating, and abdominal pain) of IBS-D patients who were treated with donor FMT were correlated with the change in uroguanylin immunoreactive cell density in the duodenum compared with controls [148]. In addition, Sun et al. [149] found FMT could alleviate small intestinal transit and reduce the concentration of DCA and CA in the high-fat diet (HFD-) fed rat model, which may be involved in downregulating the expression level of TPH1 and reducing the concentration of serotonin in the gut. On the other hand, some evidence has shown that FMT had no obvious therapeutic effect on IBS. In 2019, a systematic review and meta-analysis has shown there was no significant difference in improving the symptoms of IBS after 12 weeks of FMT treatment compared with placebo (RR 5 0.93; 95% CI 0.48–1.79) and had lower evidence in the grading of recommendation development, assessment, and evaluation quality of the body [150]. Moreover, 59.5% (95% CI 49.1–69.3) of IBS patients showed an obvious improvement of the symptoms in single-arm trials, while there was no significant improvement in RCTs compared to control (RR1/4 0.93 (95% CI 0.50–1.75)) [151]. Additionally, studies reported that it was too limited to draw sufficient conclusions depending on the current data on FMT in treating IBS and might occur some adverse reactions during treating with FMT, including Gram-negative bacteremia, death, and perforation/tear [152–154].

### 5.3. Cholestyramine

As what has been discussed above, about 68% of patients with IBS-D have abnormal bile acid absorption, of which 10% have severe idiopathic bile acid malabsorption (IBAM) (SeHCATretention < 5%), 32% have moderately severe I-BAM (SeHCAT < 10%), and 26% have I-BAM at SeHCATretention < 15%[29], and they could increase the synthesized and excreted levels of BAs compared to the patients with IBS-C or healthy volunteers [155]. In 2016, the American Gastroenterological Association (AGA) has recommended bile salt sequestrants as one of the effective drugs in the treatment of IBS-D [65]. It could increase the fecal BA excretion via regulating the enterohepatic bile acid circulation, thereby upregulating the synthesis of BAs in the liver [156]. The most commonly used bile acid sequestrant is cholestyramine [157], which can increase cecal SCFA production and downregulate the mRNA expression level of intestinal SHP in rats [158, 159]. While SCFA takes part in the process that microbiome regulates the CNS causing the interaction between brain and gut, SHP is the downstream gene of FXR. Therefore, cholestyramine might be involved in regulating the gut microbiota-bile acid axis to treat IBS-D.

## 6. Genetic Engineering of Bacteria

Since the rapid development of microbial technology, the production of probiotics with satisfying specific needs has been a close reality. It was reported that a phase I clinical trial with transgenic Lactococcus lactis expressing mature human interleukin-10 instead of thymidylate synthase for the treatment of Crohn was successful to improve the clinical scores of these patients [160]. Moreover, Bacteroides fragilis plays a vital influence on the deconjugation process of BAs [80] and is more abundant in IBS patients [161]. The polysaccharide A (PSA-) producing Bacteroides fragilis could restore normal cytokine production by correcting the TH1/TH2 imbalances and systemic T cell deficiencies [162] and prevent colitis in mouse model induced by 2,4,6-trinitrobenzenesulfonicacid (TNBS) [163]. Although the application of bacterial genetic engineering in IBS has not been reported in the literature, considering the close relationship between intestinal microbiota and IBS, it may be a potential and promising treatment strategy.

## 7. Conclusions and Perspective

There is considerable and growing evidence indicating the significance of interactions between gut microbiota and BAs in patients and animal models with IBS-D during recent years, but still, many blind spots about gut microbiota-bile acid axis with IBS-D should need to be explored. In this review, we have highlighted the intricate connection among bile acids, gut microbiota, and IBS-D, including summarizing the changes of gut microbiota in IBS-D patients, the effects of changing the gut microbiota on BA synthesis and metabolism, and the possible pathogenesis of BAs and their receptors involved in IBS-D. Moreover, given the substantial preclinical evidence for both top-down and bottom-up signaling within the gut microbiota-bile acid axis and the latest findings from clinical researches, it is a promising way to develop BA signaling and microorganisms as a target for the treatment of IBS-D.

## Figures and Tables

**Table 1:** Summary of the alterations of the gut microbiota relating to the bile acid metabolism in IBS.

Bacteria	Reactions	Percentage in IBS	Citations
Lactobacillus	Deconjugation	Lower	[35, 164–167]
Bifidobacterium		Lower	[164, 166, 168–171]
Listeria		—	[9, 172]
B. vulgatus		—	[172–174]
Bacteroides		Higher	[9, 16, 20, 47]
Clostridium		Higher	[9, 16, 47]
Enterococcus		Higher	[22, 34]

Clostridium	7*α*-Dehydroxylation	Higher	[10, 12–14]
Eubacterium		lower	[9, 34, 175]

Peptostreptococcus	Oxidation and epimerization	—	[47, 172]
Escherichia		Higher	[35, 172]
Bacteroides		Higher	[20, 176]
Clostridium		Higher	[16, 42, 49, 172, 177]
Eubacterium		lower	[34, 175]
Eggerthella		—	[172]
Ruminococcus		Higher	[172, 178, 179]
Bifidobacterium		lower	[47, 178]
Lactobacillus		Lower	[35, 47, 164, 165–167]

Clostridium	Desulfation	Higher	[47, 180, 181]
Peptococcus		Higher	[22, 47, 182]
Fusobacterium		Lower	[47, 175, 183]
Proteobacteria		Higher	[184, 185]
Pseudomonas		—	[172]

Bacteroides	Esterification	Higher	[34, 176]

Methanogens	Others	Lower	[186, 187]
Veillonella		Higher	[5, 178, 184]
Faecalibacterium		Lower	[5, 184]
Lachnospiraceae		Higher	[184]
Actinobacteria		Lower	[184, 185]
Enterobacter		Higher	[35]
Erysipelotrichaceae		Lower	[178]

Abbreviations: IBS: irritable bowel syndrome.
